# Development and characterization of low α-linolenic acid *Brassica oleracea* lines bearing a novel mutation in a ‘class a’ *FATTY ACID DESATURASE 3* gene

**DOI:** 10.1186/s12863-014-0094-7

**Published:** 2014-08-29

**Authors:** Stacy D Singer, Randall J Weselake, Habibur Rahman

**Affiliations:** 1 Department of Agricultural, Food and Nutritional Science, University of Alberta, Edmonton T6G 2P5, Alberta, Canada

**Keywords:** Low linolenic acid, Brassica oleracea, fatty acid desaturase 3 (FAD3), EMS mutagenesis

## Abstract

**Background:**

Traditional canola (*Brassica napus* L.; AACC, 2*n* = 38) cultivars yield seed oil with a relatively high proportion of α-linolenic acid (ALA; C18:3^*cis∆*9,12,15^), which is desirable from a health perspective. Unfortunately, due to the instability of this fatty acid, elevated levels also result in oils that exhibit a short shelf life and problems associated with use at high temperatures. As a result, the development of cultivars bearing reduced amounts of ALA in their seeds is becoming a priority. To date, several low ALA *B. napus* cultivars (~2-3% ALA of total fatty acids) have been developed and molecular analyses have revealed that the low ALA phenotype of lines tested thus far is a result of mutations within two ‘class b’ *FATTY ACID DESATURASE 3* (*FAD3*) genes. Since *B. napus* possesses six *FAD3* genes (two ‘class a’, two ‘class b’ and two ‘class c’) and ALA levels of approximately 2-3% remain in these low ALA lines, it is likely that the mutation of additional *FAD3* genes could further decrease the content of this fatty acid.

**Results:**

In this study, we generated low ALA (≤2%) lines of *B. oleracea*, which is the C genome progenitor species of *B. napus*, via ethyl methanesulphonate (EMS) mutagenesis. We identified a novel nonsense mutation within the ‘class a’ *FAD3* gene (*BoFAD3-2*) in these lines, which would result in the production of an encoded protein lacking 110 amino acids at its C terminus. When expressed in *Saccharomyces cerevisiae*, this mutant protein exhibited a drastic decline in its Δ-15 desaturase activity compared to the *wild-type* (*wt*) protein. Furthermore, we demonstrated that the expression of the mutant *BoFAD3-2* gene was significantly reduced in developing seeds of low ALA lines when compared to expression in *wt* plants.

**Conclusions:**

Given the additive nature of *FAD3* mutations on ALA content and the ease with which *B. napus* can be re-synthesized from its progenitor species, the mutant isolated here has the potential to be used for the future development of *B. napus* cultivars exhibiting further reductions in ALA content.

## Background

Canola (*Brassica napus* L., AACC, 2*n* = 38) is an amphidiploid species bearing A and C genomes derived from the parental species *B. rapa* (A genome, *n* = 10) and *B. oleracea* (C genome, *n* = 9) [1],[2], and is one of the most important oil crops worldwide. Typically, the seed oil of traditional canola cultivars is characterized by a low amount of saturated fatty acids (FAs; ~7%) and high contents of unsaturated FAs such as oleic acid (C18:1^*cis∆*9^; ~55-60%), linoleic acid (LA, C18:2^*cis∆*9,12^; ~20%) and α-linolenic acid (ALA, C18:3^*cis∆*9,12,15^; ~10%). This relatively high level of ALA is desirable in terms of nutrition as it is an essential component of the human diet and is believed to provide various health benefits [3],[4].

Indeed, it is the proportions of the three major unsaturated FAs (oleic acid, LA and ALA) that largely determine the usefulness of this oil from both food and industrial perspectives. While high levels of ALA may confer nutritional advantages, this same FA has been found to lead to rapid oxidation and instability during frying [5],[6]. It follows then that decreasing the level of ALA in canola seeds would be of value to enhance shelf life and stability of its oil at high temperatures [7]–[9]. To this effect, the development of low ALA (≤2-3%) cultivars has recently become one of the major breeding goals for the improvement of *B. napus*[10].

In oilseeds, ALA is mainly synthesized via the stepwise desaturation of oleic acid to LA, and LA to ALA, by endoplasmic reticulum (ER)-bound fatty acid desaturases (FADs), including the Δ-12 FAD2 and Δ-15 FAD3 enzymes, respectively [11],[12]. The *B. napus* genome contains 6 *FAD3* genes, three of which are derived from the A genome and three from the C genome [13],[14]. These six genes (termed *BnaA.FAD3.a*, *BnaA.FAD3.b*, *BnaA.FAD3.c*, *BnaC.FAD3.a*, *BnaC.FAD3.b* and *BnaC.FAD3.c*) can be phylogenetically grouped into three classes (a, b and c), with each class comprising a single gene from the A and C genomes, respectively [14].

To date, several low ALA (approximately 2-3%) *B. napus* lines harbouring mutations within *FAD3* genes have been characterized. Intriguingly, in every case, these mutations were apparent within only the ‘class b’ *FAD3* genes, comprising two of the six *FAD3* genes present in this species (*BnaA.FAD3.b* and *BnaC.FAD3.b*) [10],[14],[15]. Moreover, the mutations themselves were always identical, with *BnaA.FAD3.b* exhibiting a C to T substitution in the third position of the sixth codon in the seventh exon resulting in a missense mutation, and *BnaC.FAD3.b* bearing a G to A transition in the 5′ splice donor site of the sixth intron resulting in abnormal splicing. When combined, these two *FAD3* mutations exhibited a cumulative effect on the reduction of ALA content [14], which insinuates that mutations in additional *B. napus FAD3* genes will be key for obtaining further reductions in ALA within seed oil.

The alteration of ALA content to extremely low levels in *B. napus* through direct mutagenesis of this species would be a very complex endeavor as the trait is controlled by at least six gene loci (three from each of the A and C genomes). Since this would necessitate the use of an enormously large mutagenized population, we instead chose to carry out this study on one of its parental species for ease of manipulation due to the fact that it carries only half of the gene loci. Lines of the parental species exhibiting the desired mutant gene(s) can subsequently be utilized in *B. napus* breeding either through the re-synthesis of this species [16] or interspecific crossing with *B. napus*[17],[18].

We previously isolated two *FAD3* transcripts from the seeds of *B. oleracea* and characterized low ALA lines bearing a missense mutation in the *BoFAD3-1* coding region (corresponding to *B. napus BnaC.FAD3.b*) that resulted in a significant reduction in the desaturase activity of its encoded enzyme [19]. While the mutation itself yielded a novel allele, it occurred in the gene corresponding to the previously identified ‘class b’ C genome *FAD3* gene in which a mutation at a different position was identified in low ALA lines of *B. napus*[10],[14],[15]. In this study, we isolated low ALA (approximately 2%) *B. oleracea* lines derived from ethyl methanesulphonate (EMS) mutagenesis and identified a nonsense mutation within the *BoFAD3-2* gene (corresponding to *B. napus BnaC.FAD3.a*) that drastically reduced its enzymatic activity. To the best of our knowledge, this is the first instance in which a mutation in a Brassica ‘class a’ *FAD3* gene has been linked to a low ALA phenotype. This novel mutant has the potential to be of use in the breeding of *B. napus* to produce seed oil bearing less than 2-3% ALA through its combination with previously identified mutant *FAD3* alleles.

## Results

### EMS treatment and generation of M_1_ plants

A total of 600 seeds were treated with 0.5% EMS. While treated seeds only demonstrated a 3% reduction in viability when compared to *wt B. oleracea*, EMS treatment had a significant effect on M_1_ plants (Table 1). Indeed, approximately 30% of M_1_ plants failed to produce seed, with M_1_ plants producing an average of 29 seeds/plant and *wt B. oleracea* producing approximately 90 seeds/plant.

**Table 1:** **Treatment of** ***Brassica oleracea*** **var.** ***alboglabra*** **seeds with 0.5% ethyl methanesulphonate (EMS) and production of M**_**2**_**seeds**

**Treatment**	**No seeds used**	**No. seeds germinated**	**% germinated seeds**	**No. seedlings to soil**	**No. M**_**1**_**plants produced seeds**	**% M**_**1**_**plants produced seeds**	**No. seeds on M**_**1**_**plants**		**No. M**_**2**_**seeds harvested**
							**Range**	**Mean ± S.E.**	
0.5% EMS	600	575	95.8	450	287	63.8	1 – 512	29.2 ± 2.9	8,383
Control	120	118	98.3	10	9	90.0^a^			

^a^Number of seeds per plant was ≈ 90.

### Selection of a low ALA phenotype in M_2_ to M_7_ generations

**Figure 1:**
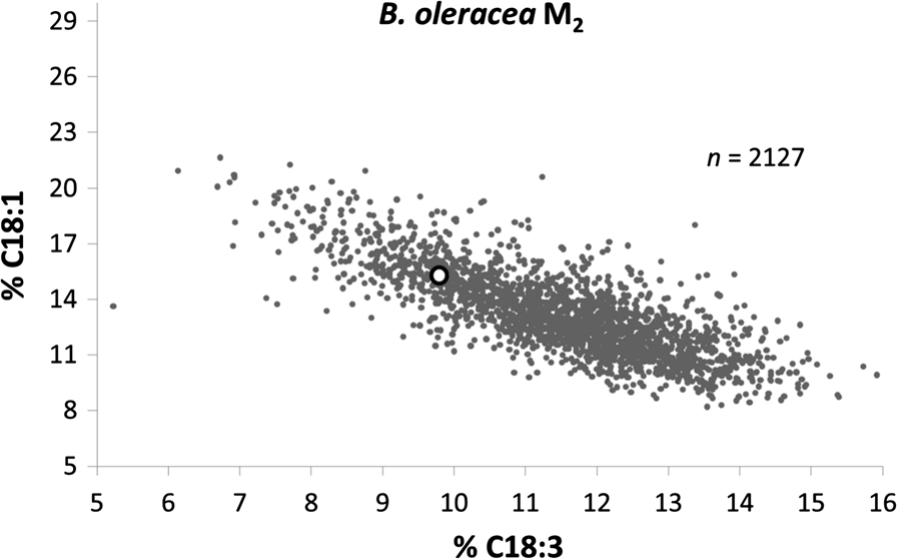
**Scatter diagram of percent α-linolenic acid (C18:3) plotted against oleic acid (C18:1) in the seed oil of an M**_**2**_**population of*Brassica oleracea*var.*alboglabra*generated from seed mutagenized with 0.5% ethyl methanesulphonate (EMS).** Open circle = *wt B. oleracea* control.

**Figure 2:**
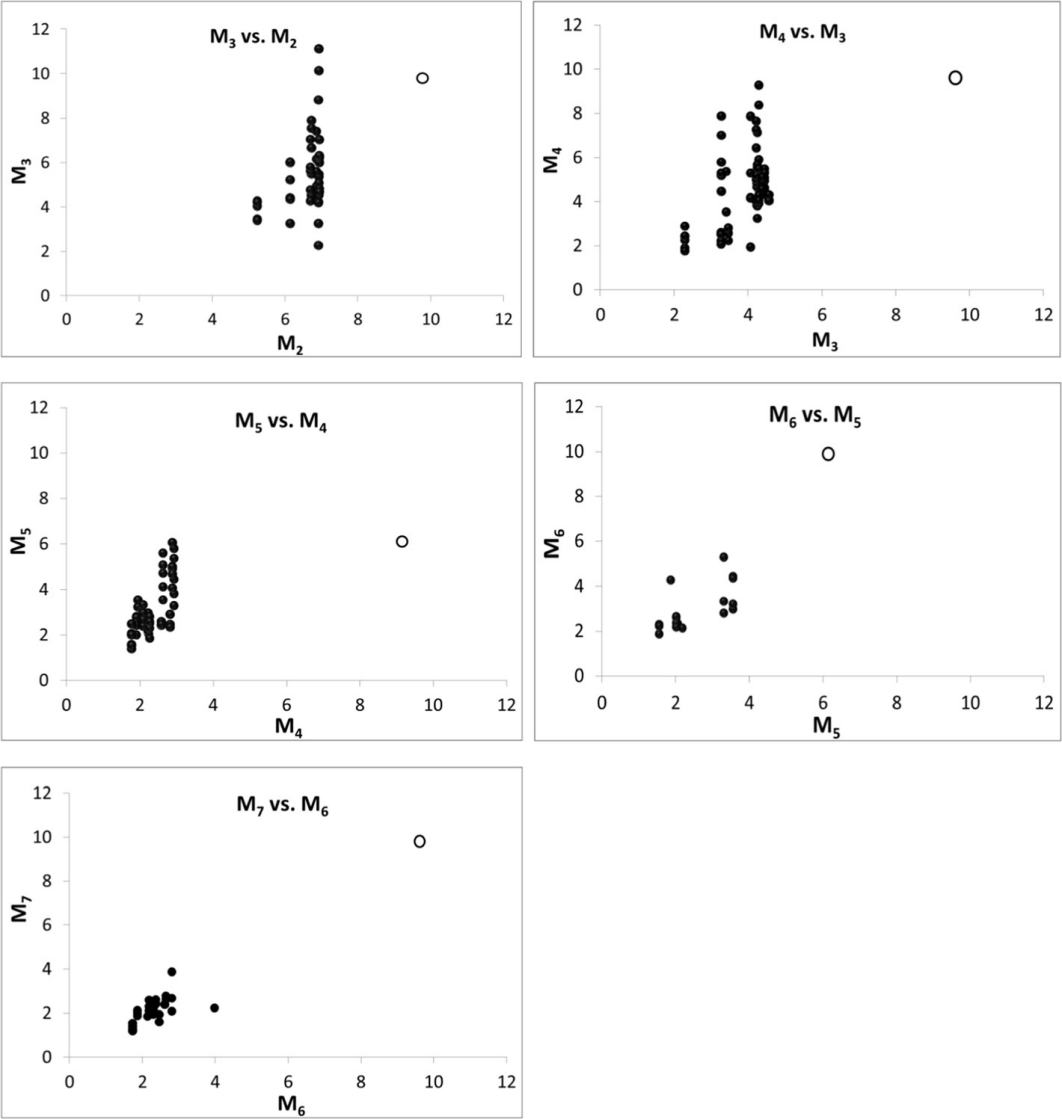
**Scatter diagrams of percent α-linolenic acid (C18:3) in seed oil derived from M**_**3**_**to M**_**7**_**generations of*Brassica oleracea*var.*alboglabra*generated from seed mutagenized with 0.5% ethyl methanesulphonate (EMS).** Open circle = *wt B. oleracea* control.

Of a total of 4,759 M_2_ plants transplanted to field plots, 97.5% produced seeds under open-pollination. Mature seeds harvested from 2,127 M_2_ plants (i.e. M_3_ seeds) were used for FA analysis. The proportion of ALA in this population varied between 3.89-15.92% (mean 11.48% ± 0.03 SE) of total FA content, while the seeds of *wt B. oleracea* contained 8.44-11.55% ALA (mean 10.15% ± 0.14 SE) (Figure 1, Additional file 1: Table S1). Of the 2,127 M_3_ seed families, 304 (14.3%) had a significantly lower ALA content than *wt B. oleracea* (confidence limit, CL_0.05_ = 9.78-10.52%). M_3_ generation plants were grown from 9 M_3_ seed families each bearing less than 7% ALA. The ALA content in seeds harvested from these M_3_ plants ranged from 2.28 to 11.13% (mean 5.55% ± 0.25 SE), which was significantly lower than *wt B. oleracea* (Figure 2, Additional file 1: Table S1). Further selection for low ALA content resulted in genetically stable low ALA mutant M_7_ lines with ≤ 2% ALA (Figure 2, Additional files 1 and 2).

When compared to *wt B. oleracea* grown under the same conditions, M_7_ lines not only exhibited a 6.5% reduction in ALA, but also a similar increase (6.9%) in the content of oleic acid. Conversely, the levels of LA in these lines were comparable to those in *wt B. oleracea* (Additional file 2: Table S2). No significant differences were noted between *wt* and mutant lines with respect to other FAs, such as C12:0, C14:0, C16:0, C18:0, C20:0, C20:1, C22:0, C22:1, C24:0, and C24:1 (Additional file 2: Table S2).

### Low ALA mutant lines possess a single nonsense point mutation in the *BoFAD3-2* coding region and reduced transcript levels compared to *wt*

**Figure 3:**
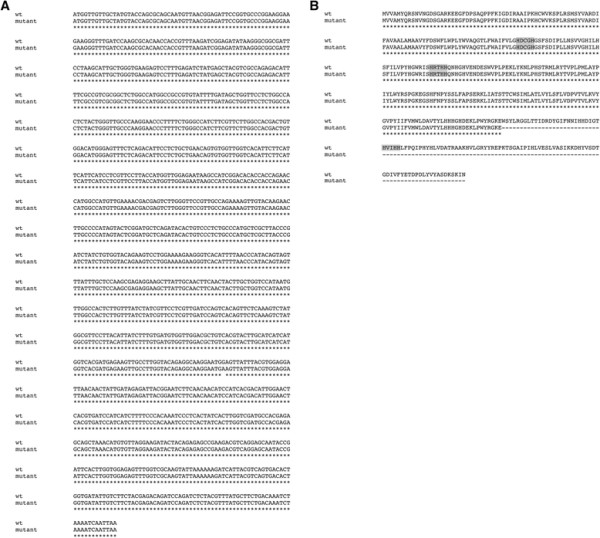
**Nucleotide (A) and amino acid (B) alignments of*BoFAD3-2*from*wt*and low α-linolenic acid (C18:3) mutant*B. oleracea*var.*alboglabra*lines.** ‘*’ indicates identical nucleotide or amino acid residues. Histidine boxes within the amino acid sequence are denoted by grey shading while the C-terminal dilysine ER-retrieval motif is outlined by a dashed box.

In order to determine whether the low ALA phenotype in mutant lines was associated with one or more mutations within either the *BoFAD3-1* or *BoFAD3-2* coding regions, *wt* sequences were compared with those isolated from two low ALA EMS mutant lines. Interestingly, while the *BoFAD3-1* sequences were identical in *wt* and mutant lines, *BoFAD3-2* contained a single nucleotide substitution from G to A (Figure 3A) at position +822 (where +1 corresponds to the first nucleotide of the translational start codon) in both mutant lines compared to *wt*. This site lies within the putative seventh of eight exons of the *BoFAD3-2* gene and results in the conversion of a TGG codon (encoding tryptophan) to a TGA stop codon. This mutation would correspond to premature translational termination and a deduced truncated protein product of 273 amino acids versus the deduced *wt* BoFAD3-2 protein of 383 amino acids (Figure 3B).

**Figure 4:**
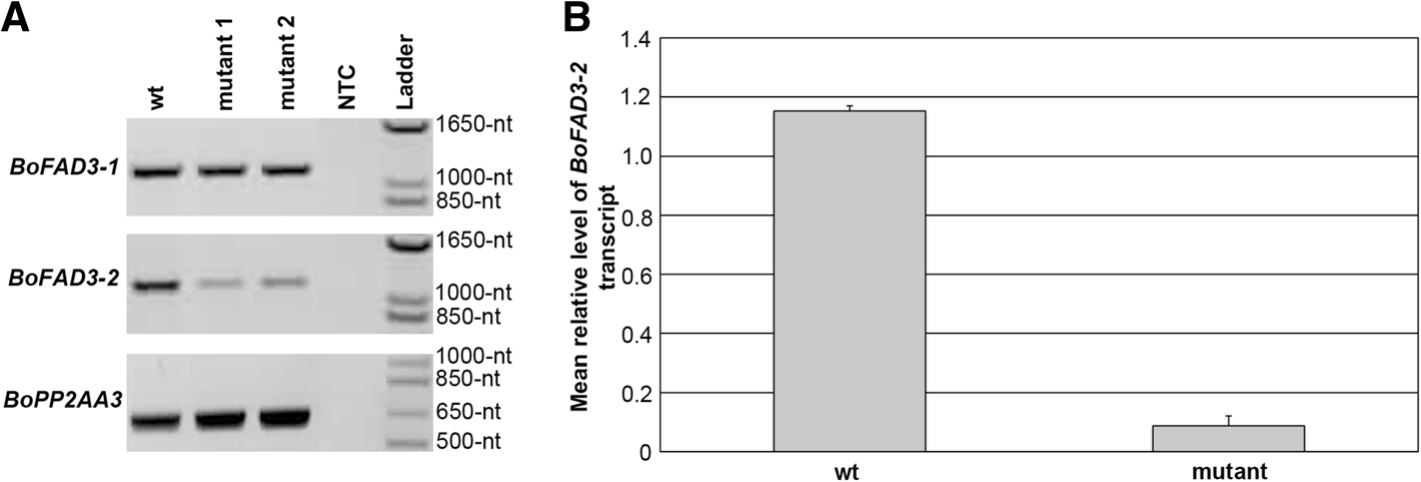
**Expression of*BoFAD3*genes in*wt*and low α-linolenic acid (C18:3) mutant*B. olereacea*var.*alboglabra*lines.(A)** Semi-quantitative RT-PCR of *BoFAD3-1* and *BoFAD3-2* expression in developing siliques (25–30 days after pollination, DAP) from *wt* and two low C18:3 mutant lines. *BoPP2AA3* expression was analyzed as an internal control. **(B)** Quantitative real-time RT-PCR analysis of *BoFAD3-2* using DNA-free total RNA derived from developing siliques (25–30 DAP) from two *wt* and low C18:3 mutant lines, respectively. Each block represents the relative mean level of *BoFAD3-2* transcript normalized to the constitutively expressed internal control, *BoGAPDH*, from two biological and three technical replicates. Bars denote standard deviations. NTC, no template control; Ladder, molecular weight ladder.

Semi-quantitative RT-PCR was carried out to determine whether any differences in expression levels or splice variants of *BoFAD3-1* and/or *BoFAD3-2* existed within the mutant lines compared to *wt*. While no variations in splicing were noted between *wt* and low ALA mutant lines in either gene, both low ALA mutant lines tested appeared to possess lower levels of *BoFAD3-2*, but not *BoFAD3-1*, transcripts than *wt* (Figure 4A). Subsequent quantitative real-time RT-PCR assays confirmed this apparent reduction in *BoFAD3-2* transcript levels in low ALA mutant lines compared to *wt* (Figure 4B).

### The mutant BoFAD3-2 protein exhibits reduced desaturase activity in yeast compared to *wt*

**Figure 5:**
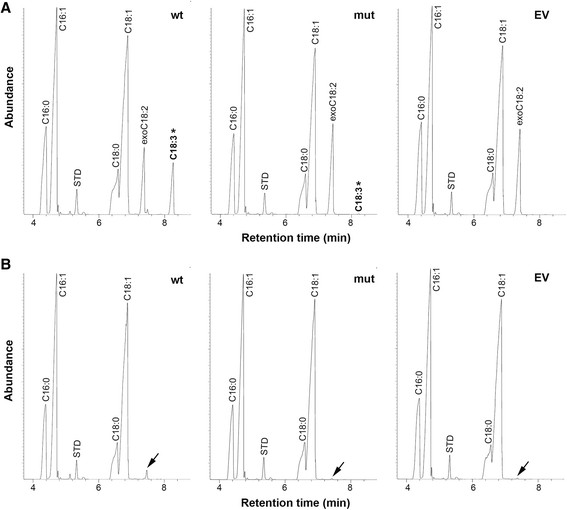
**Fatty acid analysis of*Saccharomyces cerevisiae*expressing*wt*and mutant*BoFAD3-2*by GC-MS.** FAMEs of total lipids from three independent colonies grown under inducing conditions in the presence **(A)** or absence **(B)** of 150 μM exogenously supplied linoleic acid (exo C18:2). Chromatograms are representative of yeast bearing either *wt BoFAD3-2* (wt), mutant *BoFAD3-2* (mut) or empty vector (EV) and display major common peaks (C16:0, C16:1, C18:0 and C18:1). The standard, C17:0 (STD), is also depicted in each case. α-linolenic acid (C18:3) generated by the *wt* and mutant *BoFAD3-2* enzymes in C18:2 supplemented cultures is designated by an asterisk. While all cultures supplied with exogenous C18:2 display a large corresponding peak (exo C18:2), only relatively small peaks of C18:2 were observed in non-fed yeast, which are indicated by an arrow.

**Figure 6:**
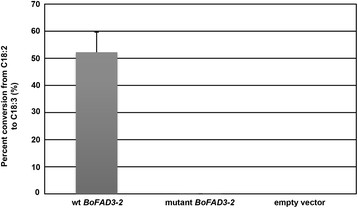
**Activity of*wt*and mutant BoFAD3-2 proteins in*Saccharomyces cerevisiae*.** FAMEs derived from three independent colonies expressing either *wt BoFAD3-2*, mutant *BoFAD3-2*, or empty vector grown under inducing conditions and supplemented with 150 μM linoleic acid (C18:2) were analyzed by GC-MS to determine desaturase activity in each case. Blocks indicate mean percent conversion from C18:2 to α-linolenic acid (C18:3) of three biological and two technical replicates. Bars represent standard deviations.

To establish whether the mutant *BoFAD3-2* coding region from low ALA lines encoded a protein with reduced Δ-15 desaturase activity compared to the *wt* sequence, both *wt* and mutant *BoFAD3-2* coding regions were expressed in *S. cerevisiae*. Supplementation of the transformed yeast cultures bearing *wt BoFAD3-2* with LA led to the production of ALA at an average level of 5.76% ± 0.98 SD of the total FA content. In contrast, yeast containing the mutant *BoFAD3-2* sequence produced only extremely low levels of ALA when supplemented with LA (0.00267% ± 0.00032 SD) while yeast bearing empty vector produced no ALA in any case (Figure 5A). The average conversion rate in LA-supplemented cultures expressing *wt BoFAD3-2* was 52.25% ± 7.36 SD, compared to a 0.025% ± 0.003 SD conversion rate in cultures expressing the mutant *BoFAD3-2* (Figure 6). Neither yeast transformed with empty vector, nor transformed yeast expressing either of the two *BoFAD3* variants that were not supplied with exogenous LA, produced any detectable ALA (Figure 5B). Furthermore, yeast fed with exogenous LA and expressing the mutant *BoFAD3-2* coding region contained significantly increased levels of LA (10.81% ± 0.31 SD) than yeast bearing the *wt BoFAD3-2* sequence (5.23% ± 0.67 SD).

Interestingly, although cultures that were not supplemented with exogenous LA did not produce any detectable ALA, yeast containing the *wt BoFAD3-2* not supplemented with LA generated significantly higher levels of endogenous C18:2 (0.85% ± 0.16 SD of total FA content) than non-fed cultures bearing either the mutant *BoFAD3-2* or empty vector (0.04% ± 0.04 SD and 0.02% ± 0.03 SD of total FA content, respectively), which were not significantly different from one another (Figure 5B). This latter result was likely due to the Δ-15 desaturation of endogenous oleic acid (C18:1^*cis∆*9^) to C18:2 ^*cis∆*9,15^ by the *wt* BoFAD3-2 enzyme, as has been reported to occur previously when both flax (*Linum usitatissimum*) and *B. napus* FAD3 enzymes were expressed in *S. cerevisiae*[20],[21].

## Discussion

As a means to enhance the stability and shelf life of canola oil, the development of *B. napus* cultivars bearing low levels of ALA in their seed oil is an important consideration for breeders. At present, several low ALA (approximately 2-3%) cultivars are available that are the result of mutations within *FAD3* genes [10],[22]–[24]. However, of the six *FAD3* genes present within the *B. napus* genome, low ALA phenotypes have only been linked to mutations within the two ‘class b’ *FAD3* genes as of yet (*BnaA.FAD3.b* and *BnaC.FAD3.b*) [14],[15],[25]. Since *FAD3* mutations appear to have an additive effect on the reduction of ALA content in various plant species [14],[26],[27], the isolation of low ALA lines resulting from mutations in additional classes of Brassica *FAD3* genes would be valuable for the future production of lines bearing further reductions in this FA. In this study, we generated and analyzed low ALA (≤2%) EMS mutant lines of *B. oleracea*, which is one of the progenitor species of *B. napus* (C genome), and identified a nonsense mutation within the ‘class a’ *BoFAD3-2* gene (corresponding to *BnaC.FAD3.a*). This is the first instance in which a mutation within a ‘class a’ Brassica *FAD3* gene [14] has been linked to low ALA content.

Low ALA content in the seeds of mutant *B. napus* lines has previously been attributed to a C to T substitution resulting in a missense mutation in *BnaA.FAD3.b* and a G to A transition in the 5′ splice donor site of the sixth intron resulting in an impairment in splicing in *BnaC.FAD3.b*[14],[15],[25]. Additionally, low ALA mutant *B. oleracea* lines have been generated that bore a single missense mutation within the putative third exon of the *BoFAD3-1* gene (corresponding to *BnaC.FAD3.b*), resulting in a significant reduction in its desaturase activity [19]. While no nonsense mutations have yet been found in low ALA Brassica mutants prior to this study, *FAD3* genes exhibiting this type of mutation have been implicated in low ALA phenotypes in other plant species, such as soybean (*Glycine max*) [26]–[28] and flax [20],[29].

In addition to the identified *BoFAD3-2* mutation, analyses of the expression levels of *BoFAD3-1* and *BoFAD3-2* in the mutant low ALA *B. oleracea* lines indicated that while *BoFAD3-1* transcript levels were apparently unchanged in mutant lines compared to *wt*, *BoFAD3-2* transcripts were significantly reduced in the mutants compared to *wt* (Figure 4). A similar reduction in expression was noted previously in a mutant *FAD3* gene isolated from a low ALA line of flax [20], and was suggested to be a consequence of nonsense-mediated mRNA decay (NMD), which serves as an mRNA quality control system that identifies and degrades mRNAs containing premature stop codons in eukaryotic organisms [30]. In plants, both unusually long 3′ UTRs and an intron located at least 50-nt downstream of the stop codon often induce NMD [30],[31]. This is almost certainly also the case in this study, as the *BoFAD3-2* mutation occurs in the seventh of eight exons, with the putative exon-exon junction occurring over 130-nt downstream of the mutation. However, an additional mutation within the *BoFAD3-2* promoter region resulting in down-regulation of the gene cannot be ruled out at this point.

While one would expect a nonsense mutation occurring at a reasonable distance from the 3′ end of a gene to produce a truncated, and theoretically inactive, version of the encoded protein, it appears that the nonsense mutation within the *BoFAD3-2* gene identified in this study may not have completely abolished the desaturation activity of this enzyme (Figures 5 and 6). Although the levels of ALA generated in yeast expressing a mutant copy of the *BoFAD3-2* coding region were miniscule compared to yeast bearing a *wt* copy of the same gene (0.0027% ± 0.00032 SD vs. 5.76% ± 0.98 SD of the total FA content, respectively), yeast containing the empty vector control did not produce any ALA whatsoever. This suggests that the mutant BoFAD3-2 protein retained at least a very small portion of residual activity. FAD3 proteins are known to contain three highly conserved histidine boxes involved in the catalytic site of the enzyme that are essential for their function [12], as well as a C-terminal dilysine motif (KSKIN) required for localization to the ER and their enzymatic activity [32]. The deduced mutant BoFAD3-2 protein identified here would lack 110 amino acids of its C terminal end (of 383 amino acids total; Figure 3B), including one of the three histidine boxes and the ER-retrieval motif, which indicates that a very small level of activity may be possible even in the absence of these two domains. This contrasts with a previous finding, whereby a flax FAD3 protein lacking only 101 amino acids of its C-terminal end was found to be completely inactive when expressed in *S. cerevisiae*[20]. While it is possible that the flax and *B. oleracea* truncated FAD3 enzymes exhibited different activities, it is also feasible that the extremely low levels of activity observed here were simply below the limit of detection in the previous study.

Seeds from the low ALA *B. oleracea* mutant lines isolated in this study also retained at least some level of ∆-15 desaturase activity, as they generated approximately 2% ALA (Figure 2, Additional file 1: Table S1). While it is possible that the mutant BoFAD3-2 enzyme may have contributed a very small proportion of this ALA, it is likely that the vast majority was mainly attributable to the remaining *wt* BoFAD3 enzyme(s) present within these lines. While the *B. oleracea* genome contains three *BoFAD3* genes, we were previously able to isolate only two of the corresponding transcripts (*BoFAD3-1* and *BoFAD3-2*) from developing seeds [19], which implies that these may be the major ∆-15 desaturases within the seeds of this species. In line with this, both *BnaC.FAD3.a* (corresponding to *BoFAD3-2*) and *BnaC.FAD3.b* (corresponding to *BoFAD3-1*) transcripts have been amplified from *B. napus* previously; however, the same is not true for the third C genome *FAD3* gene, *BnaC.FAD3.c*[14]. It is also feasible that the activity of another enzyme, such as a plastidial desaturase, could be contributing to the low levels of ALA present in these lines. This appears to be the case in Arabidopsis lines bearing mutations in their single microsomal *FAD3* gene, which still retain low levels of ALA in their seeds [33],[34].

*FAD3* mutations have not only been found to be associated with low ALA content in various plant species, but also a concomitant increase in LA content [29],[35]. Since LA acts as a substrate for FAD3, the inability of a mutant *FAD3* gene to effectively convert LA to ALA results in the depletion of ALA and a buildup of LA. This phenomenon was noted in our yeast expression assays, where yeast supplemented with exogenous LA substrate and bearing the mutant *BoFAD3-2* coding region contained significantly higher levels of LA (10.81% ± 0.31 SD) compared to yeast bearing the *wt BoFAD3-2* sequence (5.23% ± 0.67 SD). Conversely, this was not the case in our low ALA *B. olearacea* mutant lines, which displayed no significant difference in LA content compared to *wt* lines (Additional file 1: Table S1). Instead, oleic acid content was significantly increased in the mutant plants. While certain single quantitative trait loci have previously been found to affect several FAs simultaneously in other plant species, such as *B. rapa*[36], soybean [27],[37], and maize (*Zea mays*) [38], the fact that a similar increase in oleic acid was not apparent in our yeast expression assays (data not shown) indicate that a second uncharacterized mutation, possibly in a *FAD2* gene, may also be present in our mutant *B. oleracea* lines.

## Conclusions

In conclusion, we have isolated low ALA (≤2%) *B. oleracea* lines bearing a nonsense mutation in the *BoFAD3-2* gene. This mutation resulted in a severe diminishment of its ∆-15 desaturase activity when expressed in *S. cerevisiae* and may also have elicited nonsense-mediated decay of the mRNA transcript within the mutant plants themselves. As of yet, this is the first mutation to be identified in a Brassica ‘class a’ *FAD3* gene that has been linked to a low ALA phenotype. Due to the additive nature of *FAD3* mutations on ALA content in plants, this novel mutant has the potential to be of great value in the future production of designer *B. napus* lines exhibiting further reductions in this FA than have been achieved previously.

## Methods

### Plant material

A self-compatible variant of *B. oleracea* var. *alboglabra* (Chinese kale; hereafter referred to as *B. oleracea*) that exhibits a spring growth habit, and thus does not require vernalization for flowering, was used in this study (original seeds obtained from Lantmännen SW Seed, Sweden). EMS mutagenesis was carried out on a S_6_ generation inbred line developed through self-pollination of single plants. The ALA content in the seed oil of this particular line was approximately 9% (see Additional file 2: Table S2 for complete FA profile).

### Mutagenic treatment

Seed mutagenesis was performed using EMS as described previously [39]. In brief, seeds were immersed in 0.5% (v/v) EMS solution for 12 h at room temperature, and were subsequently rinsed and allowed to germinate on sterilized sand in a Petri dish. The viability of EMS-treated seeds was recorded 7–10 days after plating. M_1_ generation plants were transferred to soil (Sunshine Professional Growing Mix; Sunshine Horticulture, Bellevue, USA) and grown in a greenhouse (21°C/18°C ± 3°C day/night temperature and 16 h light with an intensity of 450 μmol m^−2^ s^−1^).

### Generation of mutant population and selection of low ALA phenotype

M_1_ plants were found to produce a much smaller quantity of visible pollen than wild type (*wt*) *B. oleracea*. Therefore, in addition to being allowed to self-pollinate within isolation bags, all M_1_ plants were also self-pollinated manually in order to ensure they would produce a sufficient number of M_2_ seeds. Manual self-pollination was carried out by pollinating 10–20 newly opened flowers on each plant with pollen obtained from the same plant. M_2_ seeds were harvested at full maturity and were initially grown in a greenhouse (with conditions as described in the previous section) in 4 cm × 4 cm × 4.5 cm (L × W × D) cells for approximately 45 days, after which time the seedlings were transplanted to the University of Alberta Research Farm in Edmonton, Alberta, Canada. This research field was isolated from other Brassica crops by a distance of approximately 300 m. Following transplantation, the field was irrigated once, and crop management practices recommended for Brassica species were followed.

Open-pollinated M_3_ seeds (i.e. seeds produced on M_2_ plants) were harvested at maturity on an individual plant basis and were utilized for FA analysis. Subsequent generations (M_3_ to M_7_) were grown either in a greenhouse (conditions as described in the previous section) or in a growth chamber (20°C/15°C day/night temperature and 16 h light with a photosynthetic photon flux density of 450 μmol m^−2^ s^−1^ at the plant level) and were self-pollinated using isolation bags. Single plants were selected for low ALA content in each generation. In every case, *wt B. oleracea* was grown alongside mutant plants as a control.

### Isolation and sequence analysis of *B. oleracea FAD3* (*BoFAD3*) cDNA

Total RNA was extracted from developing siliques 25–30 days after pollination (DAP) from *wt B. oleracea*, as well as two low ALA mutant *B. oleracea* lines, using the Qiagen Plant RNeasy kit according to the manufacturer’s instructions (Qiagen, Toronto, ON). Contaminating genomic DNA was eliminated using the TURBO DNA-free system (Ambion, Life Technologies Inc., Burlington, ON) and RNA concentrations were determined using a Nanodrop spectrophotometer (Nanodrop Products, Wilmington, DE). First-strand cDNA synthesis was conducted using the SuperScript® III first-strand cDNA synthesis kit (Invitrogen, Life Technologies Inc.) with 1 μg total RNA and an oligo-dT primer. Subsequent PCR assays were carried out to amplify *BoFAD3-1* [GenBank:JX866747] and *BoFAD3-2* [GenBank:JX866748] transcripts, which had been found previously to be expressed in *B. oleracea* developing siliques [19], using Pfx50 high fidelity DNA polymerase (Invitrogen) along with primers BoFAD3-1F1Y and BoFAD3-1RY1 [19] to amplify the full-length 1143-bp *BoFAD3-1* coding region, and BoFAD3-2FY1 (5′ – GCA ATG GTT GTT GCT ATG TAC - 3′) and BoFAD3-2FY2 (5′ – AAG TTA ATT GAT TTT AGA TTT GTC - 3′) to amplify the full-length 1152-bp *BoFAD3-2* coding region. PCR cycling parameters were as follows: 94°C for 5 min, 33 cycles of 94°C for 15 s, 54°C for 30 s, and 68°C for 2 min, followed by a final extension at 68°C for 5 min (for amplification of *BoFAD3-1*) or 94°C for 5 min, 5 cycles of 94°C for 15 s, 60°C for 30 s and 68°C for 2 min, 6 cycles of 94°C for 15 s, 57°C for 30 s and 68°C for 2 min, 26 cycles of 94°C for 15 s, 54°C for 30 s and 68°C for 2 min, followed by a final extension of 68°C for 5 min (for amplification of *BoFAD3-2*). The resulting amplification products were cloned into either the pCR 4-TOPO (Invitrogen) or pGEM-T easy vectors (Promega, Madison, WI) and sequenced. In the case of *BoFAD3-1* sequences, 10 clones derived from *wt* plants and 7 clones derived from mutant plants (two separate lines) were sequenced. In the case of *BoFAD3-2* sequences, 6 clones derived from *wt* plants and 17 clones derived from mutant plants (two separate lines) were sequenced. Sequences obtained were aligned with each other using CLUSTALW [40].

To ensure that the extreme 5′ and 3′ ends of the *BoFAD3* coding regions did not exhibit any differences between low ALA and *wt* lines, 5′ and 3′ RACE were carried out as described previously [19]. In each case, purified PCR products were cloned into the pGEM-T easy vector and sequenced, and full-length *BoFAD3* coding sequences were obtained by combining the RT-PCR sequencing results with the RACE data.

### Expression analysis of *BoFAD3* transcripts in *wt* and low ALA mutant lines

To determine whether *BoFAD3* transcripts from *wt* and low ALA mutant lines exhibited any variations in splicing or expression levels, semi-quantitative RT-PCR was carried out for both *BoFAD3-1* and *BoFAD3-2* transcripts as described previously [19] using 1 μL silique-derived cDNA (25–30 DAP) isolated from *wt B. oleracea*, as well as two low-ALA mutant lines, respectively, generated as described above. Primers BoFAD3F3c and BoFAD3R5 [19] were utilized to amplify a 1108-nt *BoFAD3-1*-specific product, while BoFAD3F3b and BoFAD3R5 [19] were utilized to amplify a 1110-nt *BoFAD3*-*2*-specific product. Additionally, a 677-nt *BoPP2AA3* transcript [GenBank: DK472124] was amplified as an internal control using primers BoPP2AA3F1 and BoPP2AA3R1 [19]. The conditions for PCR amplification of *BoFAD3*-specific products were 94°C for 2 min, 28 cycles of 94°C for 15 s, 55°C for 30 s, 68°C for 1.5 min, with a final extension of 68°C for 7 min. The same general parameters were used to amplify *BoPP2AA3*-specific fragments, with the exception of the annealing temperature, which was 58°C, and the extension time, which was 1 min. PCR products were resolved on 1% agarose gels and visualized with SYBR Safe (Invitrogen).

To provide additional evidence of differences in *BoFAD3-2* expression levels between *wt* and low ALA mutant lines, quantitative real-time RT-PCR was carried out on DNA-free total RNA isolated as described above from developing siliques (25–30 DAP) of two biological replicates of *wt B. oleracea*, as well as two separate low ALA lines. First-strand cDNA synthesis was conducted using 500 ng total RNA, along with Superscript III (Invitrogen) and an oligo-dT primer in a final volume of 10 μl. Quantitative PCR assays were performed in triplicate using 1 μl of a 1/20 dilution of each cDNA as template along with SYBR green PCR master mix in a final volume of 10 μl according to the manufacturer’s instructions (Applied Biosystems, Life Technologies Inc., Burlington, ON). Assays were carried out on an ABI 7900HT Fast Real-Time PCR System (Applied Biosystems) using primers BoFAD3F3b [19] and BoFAD3-2qPCRR7 (5′ – TCT CAA AGG ACT CTT CAC CCA G - 3′) to amplify a 115-nt fragment of the *BoFAD3-2* transcript and *GAPDH*-LEFT and *GAPDH*-RIGHT [41] to amplify a 112-nt fragment of the constitutively expressed internal standard transcript, *BoGAPDH* [GenBank: EF123055]. Reactions lacking template cDNA and those without reverse transcriptase were included in each case as negative controls. Thermal parameters for amplification were 95°C for 2 min, followed by 40 cycles of 95°C for 15 s and 60°C for 1 min. Dissociation curves were generated to ascertain that only a single product was produced in each assay. Relative levels of gene expression were attained using the standard curve method and SDS v2.4 software (Applied Biosystems), with all *BoFAD3-2* expression data comprising mean values of the biological replicates normalized to those of *BoGAPDH*.

### Heterologous expression of *wt* and mutant *BoFAD3-2* in yeast

To determine the relative activities of *wt* and mutant BoFAD3-2 proteins, full-length coding regions were amplified from previously generated cDNA using the high-fidelity Pfx50 DNA polymerase along with primers BoFAD3-2F1Y and BoFAD3-2R1Y, which were designed to specifically amplify *BoFAD3-2*. The resulting PCR products were inserted downstream of the *GAL1* promoter in the pYES2.1 TOPO®TA yeast expression vector (Invitrogen) according to the manufacturer’s instructions and were confirmed by sequencing in each case. *Saccharomyces cerevisiae* strain SCY62 (*MAT* a *ADE2 can1-100 his3-11,15 leu2-3,112 trp1-1 ura3-1*) was transformed with the resulting expression vectors containing the *wt* and mutant *BoFAD3-2* coding regions, as well as empty vector, respectively, using the lithium acetate method [42].

To induce expression of the heterologous *BoFAD3-2* sequences, three separate colonies bearing each vector, including the empty vector control, respectively, were initially utilized to produce yeast pre-cultures, which were incubated at 30°C for two days in minimal media supplemented with 2% raffinose and lacking uracil. These pre-cultures were then used to inoculate 2 × 80 mL of induction media (minimal media containing 2% galactose and 1% raffinose, but lacking uracil) to an OD_600_ of 0.2. In each case, one of the two cultures was supplied with 150 μM LA (in ethanol) along with 0.1% tyloxapol, while the other was supplemented with the same volume of ethanol and tyloxapol, without FA. The cultures were subsequently grown at 18°C with shaking at 225 rpm for 3 d.

### FA analysis

FA analysis of *B. oleracea* seed oil was carried out via gas chromatography of FA methyl esters derived from mature bulk seed (0.1 – 0.25 g) harvested from M_2_ to M_7_ generation plants as described previously [19]. Determination of the FA compositions of total lipids obtained from transformed yeast bearing *wt BoFAD3-2*, mutant *BoFAD3-2*, or empty vector, was accomplished using gas chromatography/mass spectrometry as described previously [19]. In the case of yeast FA analyses, relative percentages of ALA were derived from peak areas and conversion rates of FA substrate to product were calculated by dividing the weight percent product by the sum of the weight percent substrate and product, then multiplying by 100.

### Statistical analyses

Scattered diagrams and statistical analyses including mean, variance, standard error, confidence intervals and *t*-tests were calculated using the EXCEL program. For paired 2-tailed *t*-tests, differences were considered significant at *P* ≤ 0.05.

## Competing interests

The authors declare that they have no competing interests.

## Authors’ contributions

SDS carried out molecular analyses, as well as enzyme assays, and wrote a portion of the manuscript. RJW investigated desaturase activity, contributed to the improvement of the manuscript, and secured funding for this research. HR developed the mutant lines and wrote a portion of the manuscript. All authors read and approved the final manuscript.

## Additional files

## Supplementary Material

Additional file 1: Table S1. Variation in α-linolenic acid (C18:3) content in the seed oil of M_2_ to M_7_ generations of *Brassica oleracea* var. *alboglabra* developed through mutagenesis with ethyl methanesulphonate (EMS) and selection for low C18:3 content. This file presents population size and variation in ALA content in different generations of the mutagenized plants, as well as the proportion of plants selected for growing each subsequent generation during the development of low ALA mutant lines. Click here for file

Additional file 2: Table S2. Fatty acid composition of seed oil from M_7_ ethyl methanesulphonate (EMS) mutant lines of *Brassica oleracea* var. *alboglabra*. This file displays the fatty acid composition of seed oil from M_7_ generation mutant lines as compared to *wt* plants grown in the same environment. Click here for file
