# Supplementary material for: Development and characterization of low α-linolenic acid Brassica oleracea lines bearing a novel mutation in a ‘class a’ FATTY ACID DESATURASE 3 gene
Source: BMC Genet. 2014 Aug 29;15:94. doi: 10.1186/s12863-014-0094-7 (PMC4236532; doi:10.1186/s12863-014-0094-7)
Supplement: Additional file 1: Table S1. — Variation in α-linolenic acid (C18:3) content in the seed oil of M2 to M7 generations of Brassica oleracea var. alboglabra developed through mutagenesis with ethyl methanesulphonate (EMS) and selection for low C18:3 content. This file presents population size and variation in ALA content in different generations of the mutagenized plants, as well as the proportion of plants selected for growing each subsequent generation during the development of low ALA mutant lines. [file s12863-014-0094-7-S1.docx]

**Additional file 1: Table S1.** Variation in α-linolenic acid (C18:3) content in the seed oil of M_2_ to M_7_ generations of *Brassica oleracea* var. *alboglabra* developed through mutagenesis with ethyl methanesulphonate (EMS) and selection for low C18:3 content

| Generation and EMS treatment | No. plants grown | No. plants produced seeds | % plants produced seeds | C18:3 fatty acid analysis | | | No. plants selected | % selected plants |
| --- | --- | --- | --- | --- | --- | --- | --- | --- |
|  |  |  |  | No. plants | Range | Mean ± S.E. |  |  |
| ***M_2_*** | 4,759 | 4,640 | 97.5 | 2,127 | 3.89 – 15.92 | 11.48±0.03 | 9 | 0.42 |
| *B. alboglabra* |  |  |  | 24 | 8.44 – 11.55 | 10.15±0.14 |  |  |
| ***M_3_*** | 68 | 47 | 69.1 | 47 | 2.28 – 11.13 | 5.55±0.25 | 14 | 29.8 |
| *B. alboglabra* |  |  |  | 3 | 9.68 – 10.37 | 10.10±0.21 |  |  |
| ***M_4_*** | 84 | 63 | 75.0 | 66 | 1.77 – 9.27 | 4.55±0.22 | 12 | 18.2 |
| *B. alboglabra* |  |  |  | 4 | 8.87 – 10.21 | 9.61±0.31 |  |  |
| ***M_5_*** | 72 | 58 | 80.6 | 58 | 1.39 – 6.06 | 3.07±0.15 | 7 | 12.1 |
| *B. alboglabra* |  |  |  | 4 | 5.46 – 6.35 | 5.82±0.21 |  |  |
| ***M_6_*** | 35 | 24 | 68.6 | 17 | 1.87 – 5.30 | 3.02±0.24 | 10 | 58.8 |
| *B. alboglabra* |  |  |  | 1 |  | 9.75 |  |  |
| ***M_7_*** | 27 | 27 | 100.0 | 22 | 1.85 – 3.87 | 2.38±0.06 |  |  |
| *B. alboglabra* |  |  |  | 3 | 8.33 – 9.83 | 8.86±0.49 |  |  |

Note: For fatty acid analysis, open-pollinated seeds harvested from M_2_ plants were used, while self-pollinated seeds were used in the case of all other generations
