# Supplementary material for: Development and characterization of low α-linolenic acid Brassica oleracea lines bearing a novel mutation in a ‘class a’ FATTY ACID DESATURASE 3 gene
Source: BMC Genet. 2014 Aug 29;15:94. doi: 10.1186/s12863-014-0094-7 (PMC4236532; doi:10.1186/s12863-014-0094-7)
Supplement: Additional file 2: Table S2. — Fatty acid composition of seed oil from M7 ethyl methanesulphonate (EMS) mutant lines of Brassica oleracea var. alboglabra. This file displays the fatty acid composition of seed oil from M7 generation mutant lines as compared to wt plants grown in the same environment. [file s12863-014-0094-7-S2.docx]

**Additional file 2: Table S2.** Fatty acid composition of seed oil from M_7_ ethyl methanesulphonate (EMS) mutant lines of *Brassica oleracea* var. *alboglabra*

|  |  | **C12:0** | **C14:0** | **C16:0** | **C16:1** | **C18:0** | **C18:1** | **C18:2** | **C18:3** | **C20:0** | **C20:1** | **C20:2** | **C22:0** | **C22:1** | **C24:0** | **C24:1** |
| --- | --- | --- | --- | --- | --- | --- | --- | --- | --- | --- | --- | --- | --- | --- | --- | --- |
| B. albo (n = 3) | Range | 0.04-0.06 | 0.03-0.06 | 3.08-3.16 | 0.28-0.35 | 1.12-1.22 | 16.26-17.39 | 12.05-13.30 | 8.33-9.83 | 0.98-1.01 | 5.15-5.56 | 0.60-0.70 | 0.81-1.02 | 45.82-46.91 | 0.73-0.86 | 2.01-2.09 |
|  | **Mean** | **0.05** | **0.05** | **3.13** | **0.32** | **1.16** | **16.73** | **12.75** | **8.86** | **1.00** | **5.34** | **0.64** | **0.92** | **46.21** | **0.79** | **2.05** |
|  | SE (±) | 0.01 | 0.01 | 0.02 | 0.02 | 0.03 | 0.34 | 0.37 | 0.49 | 0.01 | 0.12 | 0.03 | 0.06 | 0.35 | 0.04 | 0.02 |
| M_7_ (n = 22) | Range | 0.03-0.11 | 0.04-0.11 | 2.55-3.16 | 0.23-0.42 | 0.77-1.45 | 19.53-32.95 | 8.07-18.07 | 1.85-3.87 | 0.54-1.25 | 3.24-8.59 | 0.24-1.32 | 0.77-1.51 | 41.93-50.86 | 0.22-1.12 | 1.59-3.31 |
|  | **Mean** | **0.06** | **0.06** | **2.90** | **0.35** | **1.11** | **23.63** | **11.12** | **2.38** | **0.93** | **5.11** | **0.58** | **1.14** | **47.57** | **0.63** | **2.43** |
|  | SE (±) | 0.01 | 0.003 | 0.03 | 0.01 | 0.04 | 0.71 | 0.50 | 0.06 | 0.04 | 0.21 | 0.05 | 0.05 | 0.59 | 0.05 | 0.08 |
